# Physicians’ Strikes

**Published:** 1901-07

**Authors:** 


### Physicians' Strikes.

We have long wondered when physicians, irritated by a hundred forms of popular ingratitude, would go on a strike. While laboring heroically to prevent disease, and thus committing professional suicide, we are constantly maligned, the lowest kind of protection is generally denied to us by the lawmakers and government-protected quackery flourishes. But according to the proverb, even the worm will turn; and it has turned in Germany. The sickness bureaus (Krankenkassen) are the intermediaries whereby about 30,000,000 people obtain practically free medical service—of course at the expense of the profession. When the bureaus made the physicians into slaves by all sorts of demands, at about fifteen cents a call, or seventy-five dollars a year, most naturally there was a "strike," or what the newspapers call a recurrence of the old question of "recognizing the union." Fifteen cent fees for cases of obstetrics, or operations, was more than human nature, even of the long-suffering bureaucratic German type, could endure.

The strange thing in all these cases is that the public does not recognize that the service obtained by such methods and prices can hardly be called medical. How can therapeutics be possible under such conditions? Physicians would be more than human who could keep such ludicrous relations from vitiating into extremes of abuse and degradation. It is as poor financial policy for the patient as for the doctor. We wish that the charges of "medical monopoly" by the hate-filled antis were truer or more possible of realization among us.—*Editorial in American Medical,* May 18, 1901.

